# The Shrimp NF-κB Pathway Is Activated by White Spot Syndrome Virus (WSSV) 449 to Facilitate the Expression of *WSSV069* (*ie1*), *WSSV303* and *WSSV371*

**DOI:** 10.1371/journal.pone.0024773

**Published:** 2011-09-12

**Authors:** Pei-Hui Wang, Zhi-Hua Gu, Ding-Hui Wan, Ming-Yan Zhang, Shao-Ping Weng, Xiao-Qiang Yu, Jian-Guo He

**Affiliations:** 1 MOE Key Laboratory of Aquatic Product Safety / State Key Laboratory of Biocontrol, School of Life Sciences, Sun Yat-Sen University, Guangzhou, People’s Republic of China; 2 School of Marine Sciences, Sun Yat-Sen University, Guangzhou, People’s Republic of China; 3 Division of Cell Biology and Biophysics, School of Biological Sciences, University of Missouri-Kansas City, Kansas City, Missouri, United States of America; University of South Florida College of Medicine, United States of America

## Abstract

The Toll-like receptor (TLR)-mediated NF-κB pathway is essential for defending against viruses in insects and mammals. Viruses also develop strategies to utilize this pathway to benefit their infection and replication in mammal hosts. In invertebrates, the TLR-mediated NF-κB pathway has only been well-studied in insects and has been demonstrated to be important in antiviral responses. However, there are few reports of interactions between viruses and the TLR-mediated NF-κB pathway in invertebrate hosts. Here, we studied *Litopenaeus vannamei* Pelle, which is the central regulator of the Toll pathway, and proposed that a similar TLR/MyD88/Tube/Pelle/TRAF6/NF-κB cascade may exist in shrimp for immune gene regulation. After performing genome-wild analysis of white spot syndrome virus (WSSV) encoded proteins, we found that WSSV449 shows 15.7-19.4% identity to Tube, which is an important component of the insect Toll pathway. We further found that WSSV449 activated promoters of Toll pathway-controlled antimicrobial peptide genes, indicating WSSV449 has a similar function to host Tube in activating the NF-κB pathway. We suspected that WSSV449 activated the Toll-mediated NF-κB pathway for regulating viral gene expression. To test this hypothesis, we analyzed the promoters of viral genes and found 40 promoters that possess NF-κB binding sites. A promoter screen showed that the promoter activities of *WSSV06*9 *(ie1)*, *WSSV303* and *WSSV371* can be highly induced by the shrimp NF-κB family protein LvDorsal. WSSV449 also induced these three viral promoter activities by activating the NF-κB pathway. To our knowledge, this is the first report of a virus that encodes a protein similar to the Toll pathway component Tube to upregulate gene expression in the invertebrate host.

## Introduction

In mammals, pathogen recognition by Toll-like receptors (TLRs) is central to the activation of the innate immune response. TLRs can interact with distinct pathogen-associated molecular patterns (PAMPs) derived from viruses, bacteria and fungi. Stimulated by PAMPs, all of the TLRs, except TLR3, recruit the adaptor protein myeloid differentiation primary response protein 88 (MyD88) through the Toll-IL-1R (TIR) domain, leading to the receptor complex formation of IL-1 receptor-associated kinase 4 (IRAK4), IRAK1 and tumor necrosis factor receptor-associated factor 6 (TRAF6) [1], [2], [3]. The activation of IRAK4 and IRAK1 leads to IRAK1-TRAF6 complex dissociation from the receptor complex to further activate downstream IκB kinase (IKK) [1], [2], [3]. Subsequently, IKK phosphorylates IκB, an NF-κB inhibitory protein. Phosphorylated IκB undergoes degradation by the ubiquitin-proteasome system, thereby freeing NF-κB to translocate into the nucleus and activate expression of proin flammatory cytokine genes [1], [2], [3]. In the MyD88-independent pathway, the detection of PAMPs, like viral dsRNA by TLR3, recruits TIR domain-containing adaptor inducing IFN-β (TRIF), TRAF6 and TRAF3 to activate NF-κB and interferon (IFN) regulatory factor (IRF) 3/7 signaling for the induction of pro-inflammatory genes and type I IFNs [3].

In *Drosophila*, the recognition of Gram-positive bacteria and fungi by peptidoglycan recognition proteins (PGRPs) and Gram-negative bacteria binding proteins (GNBPs), but not Toll itself, triggers a proteolytic cascade to cleave the endogenous Toll ligand spaetzle for binding of the Toll receptor and induction of dimerization [4], [5]. Dimerization of the Toll receptor recruits a pre-existing Myd88/Tube complex that ultimately recruits Pelle and TRAF6 to form the receptor complex[6]. MyD88-Tube-Pelle can form a heterotrimeric complex through their death domains downstream of the activated Toll receptor [7], [8]. Pelle can physically and functionally interact with TRAF6 and phosphorylate TRAF6 *in vitro* [9]. Activation of Pelle leads to the degradation of Cactus (the homolog of mammalian IκB), releasing the NF-κB family protein Dorsal into the nucleus for the transcriptional induction of immune-related genes, such as antimicrobial peptides (AMPs) [4], [5], [6]. Although no component of *Drosophila* Toll pathway has been identified as the detector of viruses, certain viruses can also activate the Toll pathway and induce AMP expression [10], [11]. However, most of the knowledge regarding the invertebrate TLR signaling pathway is limited to *Drosophila*.

The evolution of the TLR-mediated NF-κB pathway is conserved in insects and mammals [12], and IRAK family proteins are central regulators of the TLR-mediated NF-κB pathway from *Drosophila* Pelle to human IRAKs [12], [13], [14]. The mammal IRAK family of proteins includes IRAK1, IRAK2, IRAK3/M, and IRAK4, which all play a crucial role in the signaling pathways initiated by the TLRs [13]. These proteins are characterized by a C-terminal protein kinase domain and an N-terminal death domain that mediates interactions with MyD88-family adaptor proteins [13]. Whereas IRAK1 and IRAK4 have kinase activity, IRAK2 and IRAK3/M are catalytically inactive [13]. IRAK4, a central element in the early signal transduction of the TLR pathway upstream of IRAK1, is the closest mammalian homolog to *Drosophila* Pelle [15]. It was believed that Pelle was the only IRAK family protein present in invertebrates. However, a recent study has proposed that Tube arose from a gene coding a protein kinase very similar in overall structure to *Drosophila* Pelle and the vertebrate IRAKs [14]. *Drosophila* Tube has an N-terminal death domain and a C-terminal Tube repeat domain. The Tube death domain acts as a bridge between the death domains of MyD88 and Pelle for protein interactions [7], [8], [14].

The TLR-mediated NF-κB pathway is often targeted by viruses to benefit infection and viral replication. A46R and A52R from vaccinia virus contain a TIR domain and interact with the host TIR-containing adaptor protein MyD88 to block the TLR-mediated NF-κB pathway [16], [17], [18]. An IκB homolog encoded by African swine fever virus and some pathogenic orthopoxviruses interacts directly with NF-κB to repress the NF-κB pathway for the downregulation of proin flammatory genes [19], [20], [21], [22]. In contrast, proteins encoded by xenotropic murine leukemia virus-related virus (XMRV), HIV-1 and Human T-lymphotropic virus Type I (HTLV-1) activate the NF-κB pathway to promote viral gene transcription and replication [23], [24], [25], [26], [27], [28]. In mammals, some viruses seem to learn how and when to switch the NF-κB pathway off and on, which is to their benefit [25], [28]. In invertebrates, there are few reports on the manipulation of the NF-κB pathway by viral pathogens. To our knowledge, only the polydnaviruses have been reported to interact with host immune signaling molecules by encoding IκB-like proteins to inhibit NF-κB activation and suppress the insect immune response, similar to the function of the insect IκB homolog Cactus [29], [30].

WSSV is one of the most common and most destructive pathogens in shrimp aquaculture, and shrimp mortality can reach 100% within 3–10 days after infection [31]. WSSV hosts include shrimp, crayfish, crabs, lobsters and copepods [31]. In addition, WSSV can replicate its genome in insect cells [32]. The complete WSSV genome is double-stranded circular DNA of approximately 300 kbp covering a total of 531 putative open reading frames (ORFs) [33]. Herein, we investigated the interactions between the shrimp TLR-mediated NF-κB pathway and WSSV infection. We first characterized LvPelle from *Litopenaeus vannamei* and proposed that a TLR/MyD88/Tube/Pelle/TRAF6/NF-κB pathway may exist in shrimp. Then, we reported that WSSV449, a viral protein shows similarity to host Tube, can function like Tube by activating the NF-κB pathway in *Drosophila* Schneider 2 (S2) cells. Lastly, we performed a promoter screen and identified the viral genes regulated by WSSV449 through the activation of the NF-κB pathway. Collectively, the results suggest that the shrimp TLR-mediated NF-κB pathway can be activated by WSSV449 for viral gene expression.

## Materials and Methods

### Microorganisms and experimental shrimp

The Gram-negative bacteria *Vibrio alginolyticus* was cultured in Luria broth (LB) medium overnight at 28°C. Then, the cells were pelleted at 5000 × g for 10 min, washed, and resuspended in 1x PBS buffer to a density of 10^7^ CFU/ml. The quantification of bacteria was measured by counting the microbial CFU/ml on LB agar plates following incubation at 30°C overnight. The WSSV inoculum was prepared as previously described [34], [35]. *L. vannamei* (∼8–10 g each) were purchased from a shrimp market in Guangzhou, Guangdong Province, China. The shrimp were cultured in a recirculating water tank system filled with seawater (∼2.5% salinity) at 24–26°C. The shrimp were fed with a commercial diet at 5% of the body weight twice per day and were cultured for at least 7 days for acclimation before experiments.

### Cloning the cDNA and genome of *LvPelle*

Shrimp total RNA (0.5 µg), isolated using an RNeasy Mini Kit (Qiagen, Germany), was reverse transcribed to cDNA as PCR templates using the PrimeScript™ First Strand cDNA Synthesis Kit (TaKaRa, China). Using the templates and degenerate primers (DPPelleF and DPPelleR, Table 1), a cDNA fragment of *LvPelle* was obtained by PCR. Based on the cDNA fragment, the full-length cDNA of *LvPelle* was obtained by 5′ and 3′-rapid amplification cDNA ends (RACE), as previously described [34], [35]. The genomic DNA sequences of *LvPelle* were obtained by PCR using genomic DNA of shrimp with the primers listed in Table 1. Genomic DNA sequences adjacent to the 5′ ends of *LvPelle* were obtained using the Genome Walker™ Universal Kit (Clontech, USA) as previously described [36]. All new sequences obtained in this study have been deposited in NCBI GenBank (http://www.ncbi.nlm.nih.gov/genbank/).

**Table 1:** PCR primers used in this study.

Primers	Primer sequences (5’-3’)
cDNA cloning	
DPPelleF*	TGTAATGGIIIGTTTGTSTGG
DPPelleR*	TCYRTRWAKCCAAATCCT
5’ RACE1	TTTGGATTACGAGCAACATCAG
5’ RACE 2	AGGGCCAGTCAAGAAAGTCAT
3’ RACE 1	GCATTCAAGCGTCCTGATGT
3’ RACE 2	TCATGGAATGCAAAGTGAGAATG
**Genomic DNA sequences**	
GPelleF1	TGCAACTTGGAAAAGCTGACAG
GPelleR1	TGAAGACCCTCTGGGATTGG
GPelleF2	GATGGTAATGGAAACAATGCTGC
GPelleR2	TAGACCACTCCAAATGCTCCTTC
GPelleF3	CAAGTGAATGAGGAGCCAACC
GPelleR3	CGAGCACCTGAACCATTGTAG
**Promoter region**	
GSP1	CATCCAATGTCAAAGCTCGT
GSP2	TCACTCACCCACTGGAATCTG
**qPCR analysis**	
LvPelle-F	TGGGTCCGTGTCCAGTGAT
LvPelle-R	ACAAACAACCACACACAAGCAG
LvEF-1α-F	GAAGTAGCCGCCCTGGTTG
LvEF-1α-R	CGGTTAGCCTTGGGGTTGAG
**Protein expression**	
pAcLvPelleF	GGGGTACCATGGAGAGTGTCGAAGAGTCCATTAC
pAcLvPelleR	GCTCTAGACACACAGCTTTGCTTCTCTATATG
pAcWSSV449	GGGGTACCATGTGCACATTAAAAACATACAAAATG
pAcWSSV449	GCTCTAGATACTCCACGCTGCTTGGAGAAG
pAcLvDorsal	CGGGGTACCCGCCACCATGGTTGTTGCCCAGCGTACTTCC
pAcLvDorsal	AAGGAAAAAAGCGGCCGCCACATATCAGAAAATATCCAAAACTTACC

*I = Inosine; S = C or G; Y = C or T; R = A or G; W = A or T; K = G or T.

### Bioinformatics analysis

Multiple sequence alignments were performed using the Clustal X 2.0 program (http://www.ebi.ac.uk/tools/clustalw2). The simple modular architecture research tool (SMART, http://smart.embl-heidelberg.de) was used to analyze the deduced amino acid sequences of LvPelle. An neighbor-joining (NJ) phylogenic tree was constructed using MEGA 4.0 software (http://www.megasoftware.net/) based on the deduced amino acid sequences of IRAK family proteins in typical species. Bootstrap sampling was reiterated 1,000 times. The death domain and protein kinase domain of LvPelle were modeled by homology using SWISS-MODEL workspace (http://swissmodel.expasy.org/workspace/) with structures of *Drosophila* Pelle death domain (PDB code: 1D2ZC) and human protein kinase domain (PDB code: 2oicA) as templates, respectively. The 3D structures of proteins were generated using PyMOL (http://www.pymol.org/).

### Real-Time quantitative PCR analysis

For the immune challenge experiments, healthy *L. vannamei* were injected intramuscularly at the third abdominal segment with 2.4×10^6^ *V. alginolyticus*, which can cause ∼90% mortality within 30 h, or with 100 µl of WSSV inoculum. Shrimp tissues were collected as previously described [34], [35]. The total RNA isolated from the collected tissues was treated with RNase-free DNase I (Qiagen, Germany) to remove contaminated genomic DNA and then reverse transcribed to cDNA for use as PCR templates. One microliter of cDNA was used to detect the expression of *LvPelle* in healthy and immune-challenged shrimp using Master SYBR Green I system and a LightCycler (Roche) as previously described [34], [36]. qPCR was conducted in three replicates per sample, and at least three shrimp were analyzed for each sample. The standard curves for *LvPelle* and *LvEF-1α* were generated by running triplicate reactions of 10-fold serial dilutions (10 different cDNA concentrations). The efficiencies for *LvPelle* and *LvEF-1α* were 2.009 and 2.023, respectively. The relative standard curve method was used for the calculation of fold changes in gene expression as previously described [34], [36].

### Plasmid construction

For protein expression in S2 cells, pAc5.1/V5-His A (Invitrogen, USA) and PCR products amplified with pAcLvPelleF and pAcLvPelleR were double digested, purified, ligated, and transformed into DH5α competent cells to select positive clones for sequencing. Using the same method, pAc5.1-WSSV499, pAc5.1-LvDorsal, pAc5.1-N-GFP or pAc5.1-LvPelle-GFP was successfully constructed with primers in Table 1 and Table S1 as previously described [34], [35]. In previous studies, we constructed luciferase reporter vectors using the following sequences: the promoter sequences of *Drosophila* AMPs, *Drosomycin* (Drs) and *Attacin A* (AttA); the *P. monodon* AMP, *Penaeidin* (containing two types of promoters, PEN453 and PEN309); and the *L. vannamei* AMP, *Penaeidin4* (PEN4) [34], [35]. Luciferase reporter genes including pGL3-PEN453, pGL3-PEN309, pGL3-PEN4, pGL3-Drs and pGL3-AttA were constructed successfully and demonstrated to be predominantly regulated through NF-κB activation [34], [35], [37], [38], [39], [40]. Using the same method, the luciferase reporter vectors of the 40 WSSV genes that have putative NF-κB binding sites in their promoters were successfully constructed with primers in Table S2 as previously described [35], [36].

### Dual luciferase reporter assays

Because no permanent shrimp cell line is available, *Drosophila* Schneider 2 (S2) cells, a hemocyte-derived cell line, were used to perform the functional and localization analysis of LvPelle [4], [5], [34], [35]. The use of S2 cells for studying the regulation of immune-related genes including AMPs through NF-κB pathway in other arthropods, especially shrimp and horseshoe crab, has been documented by numerous studies [34], [35], [37], [38], [39], [41], [42], [43]. S2 cells were maintained at 28°C in standard *Drosophila* medium (Serum-Free Medium; Invitrogen, USA), supplemented with 10% fetal bovine serum (FBS) and 1% Penicillin–Streptomycin solution (Beyotime, China). In the luciferase reporter assays, the expression plasmid (pAc5.1-Basic, pAc5.1-LvPelle, pAc5.1-WSSV499 or pAc5.1-LvDorsal), reporter gene plasmid (pGL3-Basic, pGL3-PEN453, pGL3-PEN309, pGL3-PEN4, pGL3-Drs, pGL3-AttA, pGL3-WSSV069, pGL3-WSSV303 or pGL3-WSSV371), and internal standard pRL-TK *Renilla* luciferase plasmid were co-transfected into S2 cells that were seeded into 96-well plates 24 h before transfection. S2 cells were transfected with Effectene Transfection Reagent (Qiagen, Germany). After 36 h, cells were harvested and lysed for examination of protein expression and luciferase activities using the dual luciferase reporter assay system (Promega, USA).

### Cellular localization

S2 cells were seeded onto cover slips treated with poly-l-lysine in 24-well plates. After 24 h, the cells were transfected with the constructed pAc5.1-N-GFP vectors described above. At 36 h post-transfection, the cells on the cover slips were washed twice with PBS, fixed by Immunol Staining Fix Solution, and stained with Hoechst 33258 Solution (Beyotime, China). The treated cells were observed using a Leica laser scanning confocal microscope.

### Co-immunoprecipitation

S2 cells in 60 mm plates were transfected with 0.5 µg pAc5.1-Basic and 0.5 µg pAc5.1-LvTRAF6-Myc, 0.5 µg pAc5.1-LvPelle-V5 and 0.5 µg pAc5.1-LvTRAF6-Myc, 0.5 µg pAc5.1-Basic and 0.5 µg pAc5.1-LvPelle-Myc, or 0.5 µg pAc5.1-LvPelle-Myc and 0.5 µg pAc5.1-LvTRAF6-V5. At 48 h post-transfection, the cells were lysed, and co-immunoprecipitation (co-IP) experiments were performed using the ProFound™ c-Myc Tag IP/Co-IP Kit (Pierce, USA) following the manufacturer's instructions. Briefly, cell lysates and anti-c-Myc agarose were combined and incubated at 4°C overnight with constant inversion. On the next day, the samples were applied to centrifuge spin columns and then washed three times with TBST. c-Myc-tagged proteins were eluted using non-reducing sample buffer. The elution products were analyzed by SDS-PAGE, followed by western blot using the diaminobenzidine (DAB) substrate kit (Boster, China) with anti-V5 (1∶1,000) and anti-Myc (1∶1,000) mAb.

### Statistical analysis

Student's t-test was used to compare means from two samples using Microsoft Excel wherever applicable. Multiple means comparisons were determined by a one-way ANOVA and Tukey's multiple comparison tests using SPSS 13.0 where applicable. In all cases, differences were considered to be significant at p<0.05 and to be highly significant at p<0.01. All experiments were repeated as least three times. The data are presented as the mean ± standard error (standard error of the mean, SEM).

## Results

### cDNA cloning and bioinformatics analysis of LvPelle

**Figure 1:**
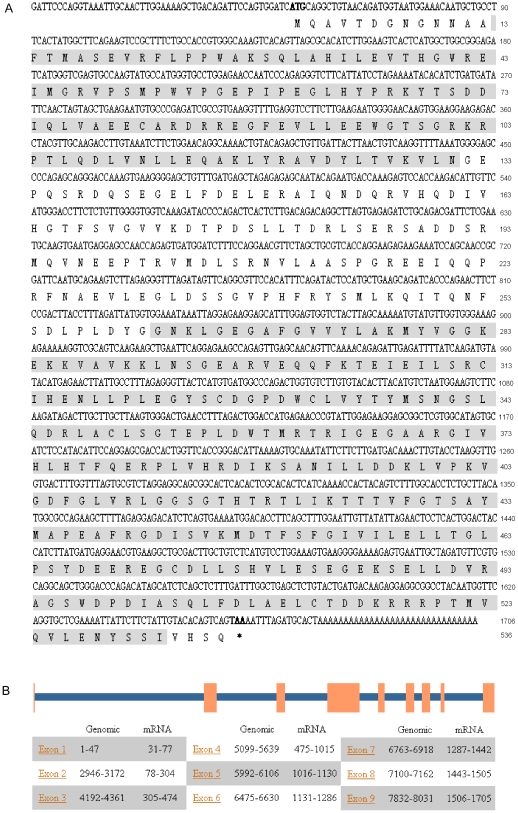
cDNA sequences and genomic structure of *LvPelle*. (A) The nucleotide (upper row) and deduced amino acid (lower row) sequences of the ORF are shown. The death domain (amino acids 14–69), and the protein kinase domain (amino acids 421–563) are shaded. (B) The genomic organization of *LvPelle*. The exons are depicted as boxes and introns as lines.

**Figure 2:**
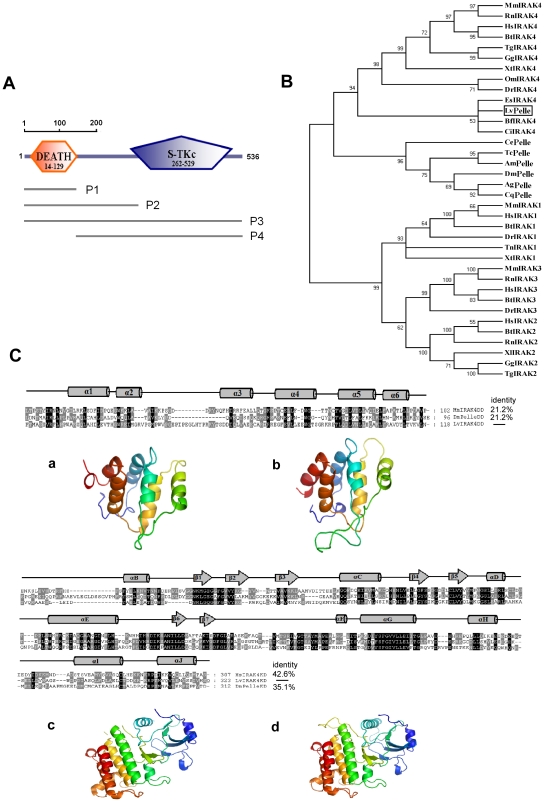
Domain architecture, phylogenetic tree and homology model of LvPelle. (A) The schematic representation of the domain topology of LvPelle. LvPelle contains an organization that is typical of IRAK family proteins: N-terminal death domain and C-terminal protein kinase domain. (B) The phylogenetic tree of LvPelle with other IRAK family proteins. The numbers at the nodes indicate bootstrap values. LvPelle is boxed. AgPelle, *Anopheles gambiae* Pelle (Accession no. **XP_311931**); AmPelle, *Apis mellifera* Pelle (Accession no. ***XP_624002***); CePelle, *Caenorhabditis elegans* Pelle/IL-1 receptor associated Kinase (IRAK) family member (pik-1) (Accession no. **NP_502587**); CqPelle, *Culex quinquefasciatus* Pelle (Accession no. **EDS41908**); DmPelle, *Drosophila melanogaster* Pelle (Accession no. **NP_476971**); TcPelle, *Tribolium castaneum* Pelle (Accession no. **XP_966383**); BfIRAK4, *Branchiostoma floridae* IRAK4 (Accession no. **XP_002601719**); BtIRAK4, *Bos taurus* IRAK4 (Accession no. **NP_001069466**); CiIRAK4, *Ciona intestinalis* IRAK4 (Accession no.** XP_002122012**); DrIRAK4, *Danio rerio* IRAK4 (Accession no. **NP_956457**); EsIRAK4, *Euprymna scolopes* IRAK4 (Accession no. **AAY27972**); GgIRAK4, *Gallus gallus* IRAK4 (Accession no. **NP_001025909**); HsIRAK4, *Homo sapiens* IRAK 4 (Accession no. **NP_001107654**); MmIRAK4, *Mus musculus* IRAK4 (Accession no. **NP_084202**); OmIRAK4, *Oncorhynchus mykiss* IRAK4 (Accession no. **CBI63176**); RnIRAK4, *Rattus norvegicus* IRAK4 (Accession no. **XP_217026**); TgIRAK4, *Taeniopygia guttata* IRAK4 (Accession no. **XP_002194205**); XtIRAK4, *Xenopus tropicalis* IRAK4 (Accession no. **NP_001116877**); BtIRAK1, *B. taurus* IRAK1 (Accession no. **NP_001035645**); DrIRAK1, *D. rerio* IRAK1 (Accession no. **XP_697688**); HsIRAK1, *H. sapiens* IRAK1 (Accession no. **AAH54000**); MmIRAK1, *M. musculus* IRAK1 (Accession no. **NP_032389**); TnIRAK1, *Tetraodon nigroviridis* IRAK1 (Accession no. **CAF93411**); XtIRAK1, *X. tropicalis* IRAK1 (Accession no. **AAH75439**); BtIRAK3, *B. taurus* IRAK3 (Accession no. **NP_001177228**); DrIRAK3, *D. rerio* IRAK3 (Accession no. **AAH98615**); HsIRAK3, *H. sapiens* IRAK3 (Accession no. **NP_009130**); MmIRAK3, *M. musculus* IRAK3 (Accession no. **AAM83393**); RnIRAK3, *R. norvegicus* IRAK3 (Accession no. **NP_001101571**); BtIRAK2, *B. taurus* IRAK2 (Accession no. **NP_001069164**); GgIRAK2, *G. gallus* IRAK2 (Accession no. **NP_001025776**); HsIRAK2, *H. sapiens* IRAK2 (Accession no. **NP_001561**); RnIRAK2, *R. norvegicus* IRAK2 (Accession no. **AAH98060**); TgIRAK2, *T. guttata* IRAK2 (Accession no. **XP_002187461**); XlIRAK2, *Xenopus laevis* IRAK2 (Accession no. **NP_001079489**). (C) Primary sequence alignments and homology models of the death domain and protein kinase domain of LvPelle. The death domain of LvPelle shows 21.2% identity to both *Drosophila melanogaster* and *Mus musculus*. The protein kinase domain of LvPelle shows 35.1% and 42.6% identity with *Drosophila melanogaster* and *Homo sapiens*, respectively. Homology models of the LvPelle death domain (b) and kinase domain (d) show high similarities with the crystal structures of *Drosophila* Pelle (a) and mammalian IRAK4 (c), respectively, providing the foundations of the evolutionarily conserved function of NF-κB signaling for LvPelle.

*LvPelle* cDNA is 1,706 bp with an ORF of 1,611 bp, a 5′ untranslated region of 50 bp, and a 3′ untranslated region of 45 bp (Fig. 1A and Fig. S1). The *LvPelle* genome is 8,031 bp containing eight exons and nine introns (Fig. 1B). The sequence was deposited in NCBI GenBank under accession no. **JN180645**. LvPelle contains N-terminal death domains and a C-terminal protein kinase domain, showing 24-40% identity with IRAK family proteins from insect to human (Fig. 2A–B). Phylogenetic trees analysis indicated that IRAK family proteins can be divided into four groups: group 1 contains vertebrate IRAK1s, group 2 contains vertebrate IRAK3s, group 3 contains vertebrate IRAK2s, and group 4 includes Pelles and IRAK4s (Fig. 2B). LvPelle is in group 4 and seems closely related to vertebrate IRAK4s. The model structures of the LvPelle death domain and protein kinase domain were generated using PyMOL Version 1.3 (Fig. 2C). LvPelle death domain consists of six α helices, which is similar to *Drosophila* Pelle and mouse IRAK4 (Fig. 2C) [44]. The protein kinase domain of LvPelle consists of nine α helices and seven β strands, which is similar to human IRAK4 (Fig. 2C) [45].

### LvPelle expression in response to pathogen infection

**Figure 3:**
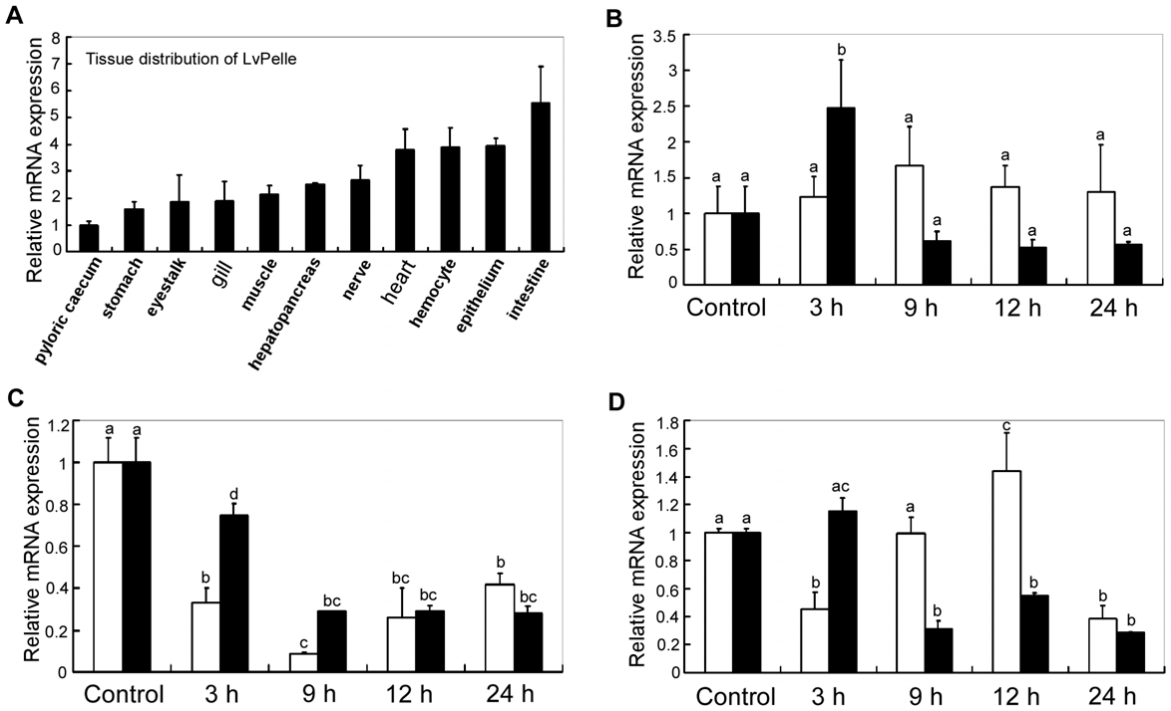
Expression of *LvPelle* in healthy and immune challenged shrimp. (A) The tissue distribution of *LvPelle* in healthy shrimp by qPCR analysis. The expression of *LvPelle* in the pyloric caecum was set to 1.0. The temporal expression of *LvPelle* in the gill (B), intestine (C) and hepatopancreas (D) after *V. alginolyticus* and WSSV challenge. The expression values were normalized to *LvEF-1α* expression values using the relative standard curve method. qPCR was conducted in three replicates per sample. Data are expressed as the mean fold change (mean ± S.E., n = 3) from the untreated group. Statistical significance was calculated by Tukey multiple comparison tests and Student's t-test. The bars with different letters indicate statistical differences (p<0.05).

In healthy shrimp, *LvPelle* was constitutively expressed in the intestine, epithelium, hemocyte, heart, nerve, hepatopancreas, muscle, gill, eyestalk, stomach and pyloric caecum (Fig. 3A). In the gill, *LvPelle* was upregulated to 2.5 times the control at 3 h after WSSV infection (Fig. 3B). In the intestine, the expression levels of *LvPelle* were decreased after *V. alginolyticus* and WSSV infection (Fig. 3C). In the hepatopancreas, *LvPelle* was upregulated up to 1.4 times the control only at 12 h after *V. alginolyticus* infection (Fig. 3D), but *LvPelle* expression levels were decreased at 9 h, 12 h and 24 h after WSSV infection (Fig. 3D).

### LvPelle localizes in the cytoplasm of S2 cells

**Figure 4:**
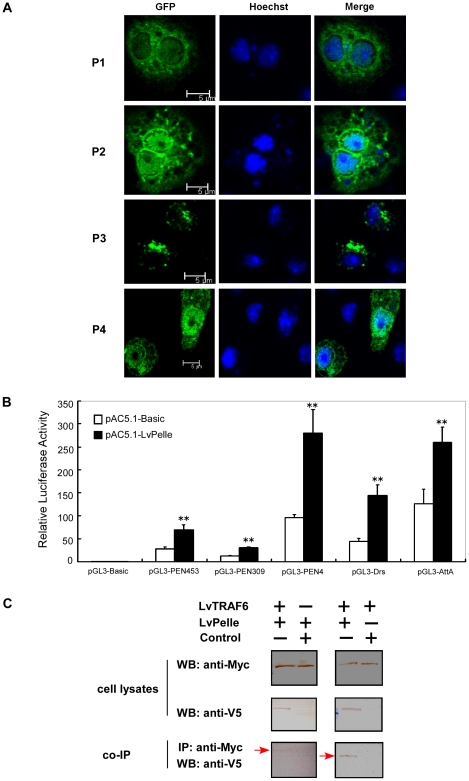
The functional study of LvPelle in the Toll pathway. (A) The intracellular localization of LvPelle and its truncated mutants fused with GFP in S2 cells. P1 represented amino acids 1–69 of LvPelle, P2 represented amino acids 1–262 of LvPelle, P3 represented amino acids 1–536 of LvPelle (the full-length protein of LvPelle) and P4 represented amino acids 129–536 of LvPelle as indicated in Fig. 2. (B) Overexpression of LvPelle activates *Drosophila* and shrimp AMP promoters. Luciferase reporter genes including pGL3-PEN453, pGL3-PEN309, pGL3-PEN4, pGL3-Drs and pGL3-AttA were constructed successfully and demonstrated to be predominantly regulated through NF-κB activation [34], [35], [37], [38], [39], [40]. In this study, we use these luciferase reporter genes to investigate the activation of Toll-mediated NF-κB pathway. The data are representative of three independent experiments. **p<0.01. (C) LvPelle associates with LvTRAF6 during TLR signal transduction. Myc-tagged LvPelle co-precipitated with V5-tagged LvTRAF6 (lane 1) and V5-tagged LvPelle coprecipitated with Myc-tagged LvTRAF6 (lane 3). pAc5.1-Basic were used as controls.

The fluorescent imaging of GFP-labeled partial or full-length LvPelle (Fig. S2) analyzed under confocal microscopy indicated that only the full length LvPelle had a correct cellular localization, in which LvPelle appeared as point-like aggregates in the cytoplasm (Fig. 4A). However, the GFP-labeled partial LvPelle was ubiquitously distributed in S2 cells (Fig. 4A). The data indicated that the LvPelle-GFP fusion protein was localized in the cytoplasm of S2 cells, which was consistent with the putative function of LvPelle in forming a receptor complex with MyD88 and Tube downstream of the TIR domain of TLRs.

### LvPelle activates *Drosophila* and shrimp AMP promoters

Here, we investigated whether LvPelle, a potential positive regulator of the Toll pathway, could activate the promoters of NF-κB pathway-controlled AMPs. In S2 cells, LvPelle can activate the promoters of both shrimp and *Drosophila* AMP genes. Expression of LvPelle induced *P. monodon Penaeidin* promoter activity by ∼2.51-fold and by ∼2.49-fold for type453 and type309 (two different promoters of *P. monodon Penaeidin*), respectively, and increased *L. vannamei Penaeidin4* promoter activity by ∼2.92-fold (Fig. 4B). LvPelle also increased the activity of *Drosophila Attacin A* and *Drosomycin* promoters by ∼2.05- and ∼3.24-fold, respectively (Fig. 4B).

### LvPelle interacts with LvTRAF6

To verify whether LvPelle could associate with LvTRAF6, co-IP assays were conducted. The results showed that Myc-tagged LvPelle coprecipitated with V5-tagged LvTRAF6 and V5-tagged LvPelle coprecipitated with Myc-tagged LvTRAF6 that (Fig. 4C), suggesting that LvPelle can associate with LvTRAF6 during TLR signal transduction.

### WSSV499 shows similarity to host Tube and activates the NF-κB pathway

**Figure 5:**
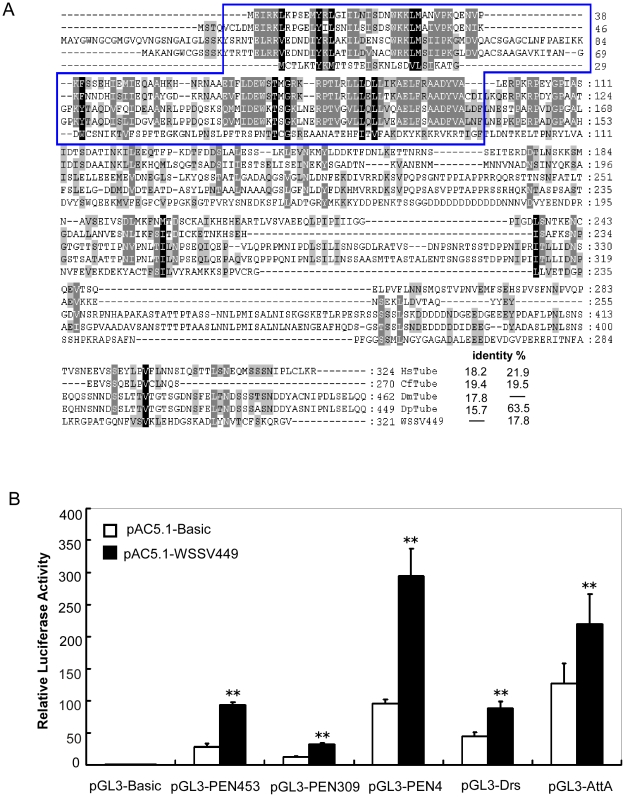
WSSV449 shows similarity to host Tube and activates the promoters of *Drosophila* and shrimp AMPs. (A) Multiple sequence alignment of WSSV449 and insect Tube proteins. The conserved functional death domain is framed with a green line. The overall protein identities are indicated. WSSV449 shows 15.7-19.4% identity to Tube, which is a positive regulator of insect Toll pathway. CfTube, *Camponotus floridanus* (Accession no. **EFN72687**); DmTube, *Drosophila melanogaster* (Accession no. **AAA28994**); DpTube, *Drosophila persimilis* (Accession no. **EDW34648**); HsTube, *Harpegnathos saltator* (Accession no. **EFN87560**); WSSV449, white spot syndrome virus 449 (Accession no. **AAL89317**). (B) Overexpression of WSSV449 activates *Drosophila* and shrimp AMP promoters. The data are representative of three independent experiments. **p<0.01.

Viruses often encode proteins similar to components of host immune pathways to control immune responses. In the 531 WSSV ORFs, we found that WSSV449 shares 15.7–19.4% identity with inset Tube, a positive regulator of the Toll pathway (Fig. 5A). To test whether WSSV449 can participate in the host Toll pathway like Tube, we performed dual luciferase reporter assays in S2 cells. We observed that WSSV449 can activate the AMP reporters of *Drosophila* and shrimp, whose induced activities are mostly controlled through multiple NF-κB binding sites in the promoters (Fig. 5B) [34], [35]. WSSV449 activated the promoters of *PmPEN453*, *PmPEN309*, *LvPEN4*, *Drosomycin* and *Attacin A* by ∼3.27-, ∼2.55-, ∼3.07-, ∼1.98 and 1.74-fold respectively (Fig. 5B).

### WSSV gene promoters have multiple NF-κB binding sites and can be stimulated by NF-κB activation

**Figure 6:**
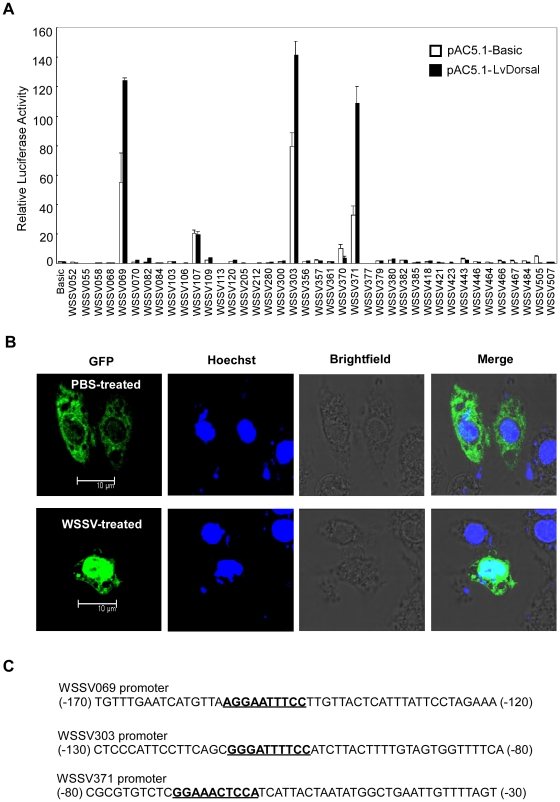
A promoter screen to identify viral genes induced by NF-κB activation. (A) The determination of the promoter activities of 40 WSSV genes when the shrimp NF-κB family protein LvDorsal is overexpressed in S2 cells. All of the 40 WSSV genes possess NF-κB binding sites in their promoter regions. The promoter regions were inserted into pGL3-Basic to construct luciferase reporters. When transfected into S2 cells, the promoters of *WSSV069* (*ie1*), *WSSV303* and *WSSV371* are activated by LvDorsal. (B) Stimulated by WSSV, LvDorsal translocated to the nucleus. PBS treated cells were used as a control. (C) The promoter regions containing NF-κB binding sites of *WSSV069* (*ie1*), *WSSV303* and *WSSV371*. The NF-κB binding sites were in bold and underlined.

**Figure 7:**
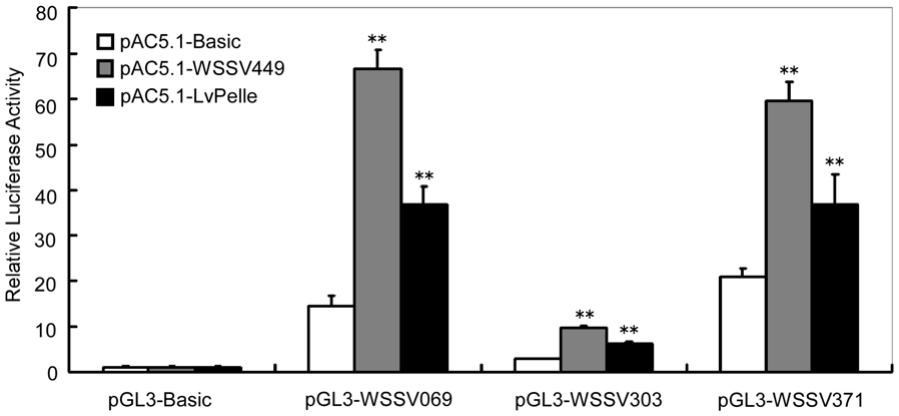
WSSV449 and LvPelle activate the promoters of *WSSV069* (*ie1*), *WSSV303* and *WSSV371*. The S2 cells were transfected with 0.05 µg of protein expression vector (pAC5.1-LvPelle or pAC5.1-WSSV449), with 0.05 µg reporter gene plasmid (pGL3-Bsic, pGL3-WSSV069, pGL3-WSSV303 or pGL3-WSSV371) and with 0.005 µg pRL-TK *Renilla* luciferase plasmid (as an internal control, Promega, USA). Thirty-six hours after transfection, the cells were harvested and analyzed using the Dual Luciferase kit. The data are representative of three independent experiments. **p<0.01.

Here, we found that 40 WSSV genes have multiple NF-κB binding sites in their promoters. To explore whether these viral genes are induced by NF-κB pathways, like certain genes of HIV, herpesviruses, hepatitis C virus (HCV) and encephalomyocarditis virus (EMCV), we performed a promoter screen [25], [28]. We observed that the promoter activities of *WSSV069 (ie1)*, *WSSV303* and *WSSV371* are highly induced compared to the control group when the shrimp NF-κB family protein LvDorsal is overexpressed (Fig. 6A). Our previous study also reported that LvDorsal could bind to NF-κB binding sites *in vitro* [39]. Stimulated by WSSV, LvDorsal can translocate to the nucleus, suggesting a transcriptional function of LvDorsal after WSSV infection (Fig. 6B). Furthermore, we observed that overexpression of LvPelle increased the promoter activities of *WSSV069* (*ie1*), *WSSV303* and *WSSV371* by ∼2.56-fold, ∼2.21-fold and ∼1.78-fold, respectively (Fig. 7). WSSV449 also activated the promoter activities of *WSSV069* (*ie1*), *WSSV303*, and *WSSV371* by ∼4.63-fold, ∼3.55-fold and ∼2.89-fold, respectively (Fig. 7). Thus, WSSV449, as well as host Pelle or Tube, can activate the NF-κB signaling pathway to regulate gene expression [8], [46], [47]. These results suggest that expression of *WSSV069 (ie1)*, *WSSV303* and *WSSV371* may depend on NF-κB signal activation.

## Discussion

The TLR-mediated NF-κB signaling pathway is essential in antibacterial and antiviral defense in *Drosophila* and humans [4], [5], [12]. This pathway also seems to be an attractive target of bacterial and viral pathogens [25], [28]. In invertebrates, however, this pathway has only been well-studied in *Drosophila,* and there are few reports of interactions between TLR signaling pathway of invertebrate hosts and viral pathogens. Here, we proposed the existence of a potential TLR/MyD88/Tube/Pelle/TRAF6/NF-κB pathway in shrimp and reported that WSSV449 showed similarity to host Tube and activated this pathway to facilitate the expression of *WSSV069 (ie1)*, *WSSV303* and *WSSV371*.

Insect Pelle or its mammalian homolog IRAK4 is the central members of the TLR-mediated NF-κB pathway because they not only form receptor complex with MyD88 and Tube (or IRAK1) but also participate in downstream signal transduction with NF-κB family proteins [13]. We found that LvPelle could function as an adapter protein in Toll signaling by activating the promoters of shrimp and *Drosophila* AMP genes, suggesting a conserved Toll signaling pathway in shrimp for AMP expression. Co-IP experiments also revealed that LvPelle could associate with LvTRAF6, a Toll pathway component downstream of LvPelle, similar to the interaction between *Drosophila* Pelle and TRAF6. Using the NCBI EST database, we found Myd88 and Tube in other crustaceans such as brine shrimp. In addition, several partial sequences of Cactus- and Myd88-like genes have been obtained in our lab. Adding to our previous report of shrimp Tolls and Dorsal and their function in participating in the Toll pathway [34], [39], [42], we proposed that shrimp possess a potential TLR/MyD88/Tube/Pelle/TRAF6/NF-κB pathway similar to *Drosophila* for immune gene regulation in antiviral and antibacterial responses [4], [5].

The WSSV genome encodes a total of 531 putative ORFs. After a homology search using NCBI BLAST, we found that several proteins show similarities with host proteins. Further sequence analysis indicated that WSSV449 shares 15.7-19.4% identity with Tube, which is a positive regulator of the insect Toll pathway [5], [8], [14], [47]. When overexpressed in S2 cells, WSSV449 highly activated the Toll pathway by inducing the promoter activities of shrimp and *Drosophila* AMPs. This result reveals that WSSV449 can activate the Toll-mediated NF-κB pathway. WSSV449 shares low identity (15.7-19.4%) with Tubes, at the same time, insect Tubes also show low identity with each other, e.g. CfTube and HsTube share only 19.5% and 21.5% identity with DmTube, respectively (Fig. 5A). So it's still possible that WSSV449, like Tube, could acts as a bridge between the death domains of shrimp MyD88 and Pelle for NF-κB activation [7], [8], [14]. An alternate possibility is that WSSV449 might interact with the death domain of shrimp MyD88, Tube or Pelle and influence the stability of shrimp MyD88-Tube-Pelle heterotrimeric complex in NF-κB activation [7], [8], [14]. To investigate the molecular mechanism of NF-κB activation by WSSV449, we are trying to obtain the full-length cDNA of shrimp MyD88 and Tube, and investigate whether WSSV449 can function as a viral Tube homolog by acting as a bridge between the death domain of MyD88 and Pelle for protein interaction.

The activation of the NF-κB pathway is a hallmark of most viral infections [25], [28]. Apart from the non-specific recognition of PAMPs by TLR, many viruses have evolved distinct strategies to control activities of the NF-κB pathway. HIV-1 proteins such as Tat, Vrp and Nef, participate in the regulation of NF-κB activity [48]. The Tax transactivator oncoprotein of HTLV-1 directly activates IKK, leading to NF-κB activation, and results in upregulation of cellular genes that promote cell proliferation and survival [24], [28], [48]. The HBx protein of hepatitis B virus (HBV) is also a potent inducer of NF-κB [25]. Some viruses, such as herpes simplex virus 1 (HSV-1), appear to activate NF-κB in a biphasic way [25]. It has been report that WSSV449 (orf390 or AAP-1) is an anti-apoptotic protein that interacts with and affects shrimp caspase [49]. Here, we found that WSSV449 also activates the NF-κB pathway. It is well known that activation of NF-κB can induce an anti-apoptotic response [50], and this raises the question of whether WSSV449-induced NF-κB activation also contributes to inhibition of cellular apoptosis.

Viruses activate the NF-κB pathway not only for blocking apoptosis and promoting cell proliferation and survival but also for stimulating viral gene expression [25], [28]. There are NF-κB binding sites in the promoters of many different classes of viruses such as HIV-1, XMRV, cytomegalovirus, herpesvirus, HBV and Epstein–Barr virus (EBV) [28]. In HIV-1-infected cells, activation of NF-κB signaling promotes long terminal repeat (LTR)-driven viral transcription. The absolute requirement for these NF-κB binding sites during the HIV-1 life cycle are required for HIV transcription in some cell types [28] NF-κB activation can also markedly increase XMRV replication through the NF-κB binding sites in the XMRV LTR. The existence of a potential TLR/MyD88/Tube/Pelle/TRAF6/NF-κB pathway in shrimp and NF-κB activation by WSSV449 make us question whether similar mechanisms are used by WSSV. After bioinformatics analysis of the promoters of the 531 putative ORFs of WSSV, we found 40 WSSV genes that have putative NF-κB binding sites in their promoters. Thus, we decided to perform a screen to identify which viral genes can be activated by NF-κB signaling. Our experiments revealed that the promoters of *WSSV069 (ie1)*, *WSSV303* and *WSSV371* can be stimulated by NF-κB activation (Fig. 6A). The shrimp NF-κB family protein LvDorsal can bind with NF-κB binding sites *in vitro* [39]. Further results confirmed that overexpression of WSSV449 can also induce the promoter activities of *WSSV069 (ie1)*, *WSSV303* and *WSSV371* by activating the NF-κB pathway in S2 cells. Therefore, it is possible that WSSV encodes proteins to activate the NF-κB pathway to facilitate viral or host gene expression, which could contribute to virus production. The roles of WSSV069 (ie1), WSSV303 and WSSV371 during WSSV infection are also of great interests. WSSV069 (ie1) is an immediate-early gene that has been reported to be a transcriptional regulator and exhibit transactivation and DNA binding activity, but its exact role in viral infection is still elusive [51], [52]. And the functions of WSSV303 and WSSV371 in viral infection are still unknown.

In summary, we report the cloning and functional study of *LvPelle*, demonstrating that an evolutionarily conserved TLR/MyD88/Tube/Pelle/NF-κB signaling pathway may exist in shrimp. We found that WSSV449 shows similarity to host Tube and activates the NF-κB signaling pathway by increasing the promoter activities of *Drosophila* and shrimp AMPs. In addition, the activation of the NF-κB signaling pathway by WSSV449 also stimulates the expression of *WSSV069 (ie1), WSSV303* and *WSSV371*, suggesting that WSSV may use mechanisms similar to HIV-1 to activate the NF-κB signaling pathway to regulate its own gene expression [25], [28], [48]. How and why WSSV449 stimulates NF-κB activity has not been clearly elucidated. Further investigations are underway in our lab to identify the targets of WSSV449, such as shrimp MyD88 or Tube, to elucidate the molecular mechanism and function of WSSV449-induced NF-κB activation.

## Supporting Information

Figure S1 The putative promoter region and genome sequence of *LvPelle*. Using the genome walker, we amplified the genomic regions upstream the 5’ end of *LvPelle* (indicated by lowercase letters). In the putative promoter region, the NF-κB, AP-1 and GATA motifs involved in the transcriptional regulation of immune genes in arthropods were identified. The putative transcription start site was in bold. The introns were shaded.
(DOC)

Figure S2 A cartoon diagram for vector constructions in cellular localization.
(DOC)

Table S1 PCR primers used in cellular localization.
(DOC)

Table S2 PCR primers used in the construction of luciferase reporter vectors of the 40 WSSV genes that have putative NF-κB binding sites in their promoters.
(DOC)
