# Supplementary material for: The Shrimp NF-κB Pathway Is Activated by White Spot Syndrome Virus (WSSV) 449 to Facilitate the Expression of WSSV069 (ie1), WSSV303 and WSSV371
Source: PLoS One. 2011 Sep 12;6(9):e24773. doi: 10.1371/journal.pone.0024773 (PMC3171479; doi:10.1371/journal.pone.0024773)
Supplement: Figure S1 — The putative promoter region and genome sequence of LvPelle. Using the genome walker, we amplified the genomic regions upstream the 5’ end of LvPelle (indicated by lowercase letters). In the putative promoter region, the NF-κB, AP-1 and GATA motifs involved in the transcriptional regulation of immune genes in arthropods were identified. The putative transcription start site was in bold. The introns were shaded. (DOC) [file pone.0024773.s001.doc]

gtgtttgtgtgtgtgtgtgtgtgtgtgtgtgtgtgtgtgtgtctgtatatgtatgtatatgcatatatatgtatacacacacacacacacatgaatgtgtgtgtgtaacagtcgtcagttaatattgcagtgactcctgtcgcctcacagacccaaatacaataaaacaataatatcaaaaggc

AP-1

tgtagagagtttgtcaaaacctccatcagcagtgtccgattcccttattacagttccgaagtgatagccacgccatcatcgctgcattcctc

GATA-1 GATA-1

tgtctactgcccttcttgattgtcacaggatcattacagaatgtgcgcaaaacagcagtaatggggaaaatgcggaccaaagttccgtcgggtgagaaacaatcatttacaatctagggaagttcatttaagcgtaatgtatattgaattttgagtttaacaaatcgctgtattatatctacaatttgattactaacaaaaccgctagtgagctccgagatgaggatgactgctcctagaaaggcagtcaaccagagaatcacagttataaactgg

GATA-1

aagaaattatatttcacgttaaatcatgtgaatttgcggttgctcttgcattcacttcagtttacagcgttcacatcacatcatcacttgttgcatcatatatcgagcagagcagcgaccctcagatataaacatatccacgatagcagaccctcttaaattttgttctctacttttccccaag

GATA-1

ttacgcctcgcaatacttacgttttctgtaattcgaactttcttatcaatgacgtcagtattacgtcacggcatacatgttttgg**G**ATTCC

NF-κB

CAGGTAAATTGCAACTTGGAAAAGCTGACAGATTCCAGTGGGTGAGTGAATTTCGCAAGAATACAAGAGCATATGTGTTTGTCAAGTGTTTATATGAGACATTAAGTGGATTTCAAAGTCAATACGAGCTTTGACATTGGATGTTAACGCCCACTTGTGTGAAAGTTGTCACCAGATATTACAAGGTTCATCATATCTGACATTTAATACATTTGTTATGGTGCAGTGGAGGCCTCGTAAGAGTATATTGAGTGAATATAATTGGTTTCTTGATTTTTCCTGTTTGCATTGGTATGAATTGTTTTGGTGAAGAATCAGAAATAAATTACATTCTCACTCATTCACAGTTGTCCTATGTCTAGAATAACAAATCTATTGGATTATGATATAAAATTTTATGTCGGCTACAATTTTGTCATTTATGTTGAGATATTATAGAAATCCTCTGTCAGTGAATACATGTTATATATAGCAAACTATTTTGTTTCAAAGTTTGCATCAGCGATATAAGAATCTTCAGACTTTAGGAAAATTTCTGATTTTCAGTTTGACATTTGTACGGATCATCATTTATTTAGATTACTTTTTTGTCACAACCTTGGCTCAAACCATACAGTATATGGTTCTTATGATAGCCTTAGGACGAATAAGAATAATAACGATAATATGGTAAACTCCTTTAATGTTGGAAGATATGTAATATATAATTAACAAAACTATACAAATTGTTCAAAATGTGTCCATATGAAGTCACACCATTCTGATATGCTTTGCTTTTTGGAAAAGCGGAAAAGGCTAGTAAATCTTCCTGATGTATATTCAGTAAAGTAGAGCTTAGAATATTTCTTTATTTATGATATATTAGTAAGGAGGTCAACAAGTTCTATCTTACTAGACTATCCATGGTAGAAGTTTGACTGCCTTTGCTGAAACACAAAATGTAGATACAGTAATGTTACTGTATTTCATTTCAGTAGCATGAATTCATAGAATCTGATGAATGGCTTTGACCGTATCTTTACTTTCTGCTGATTTTATCTCATTTTTAAAAATTTACTTTAAGGTATTTTTTTGTACTGTCTTAGTATTTGCCAGCACCATTTTTCACTATGAAATTCATGCGAGAAATCTTTTCCTGCATTTTTCTTCTCTTCACTTCCATTCCTACCTCATTCTGTTCAATATGAAAAAAAAATTATGAATGATTTTTTGTATATACATCCATTAATACAAGTTCGAATGATATATCCTGAAGAGGAATTACTTTCTGAATAATATATCTGTAGCCTCATTATTTTTTCACCATAAACATGGATGGCTCTTTTGAAATACAGTTTGTAATATGGTTAGGGCTTGTAGTTTCTGTATAATTTGTTTTAATACAGTATAGGTATTTTATTTCATAGCAGGTAGAATTAATATGCCTATTTCTCCCCTCACCCCTAGTTCATATTTTTTTCTAAGGATTGATTTTTTTCCATTATACATAATATTCTTTTTCACTGAAAGTTATGACCCCTAACCTCTTCATATATTATAAGCCTTGATTTTGGTAGACATAGTTGCTTAGTTATTTACTGTATATTTATGAGAAAGGCATTTCAGTGGTATGTAGACACTACCATGTTTTGTGATAAGAATTAAGGGCAGTTGAATAATTCAAAAATAATTATTTAAATTTGTGTTGTGACCCAGTATGGAAATATACTTCACTAGCAGATGATGTGATGAGTATAATTTTTCAATTGTTTTAGGGTATACCTACATAACGTATTATCAGCCAATTTCCATGTTCATAAGGAGTTGAAAAATAGCAATGTATTACAAAAGTCAATCATTGTTTCAAGAGTCCCTTGAAATACACTGAAATCATTAAACAAACACAGTCCTTTATTTAATGGTGTGAAATGGTGGAAGGAGATTTTAGTCCCAAATTTAAAAGAATCTAAGCAATTTCAGAGTCATATTTGAACTAAGTTTTTATGTGTTCTTATCATTTCATGTTATCATTGTATATTTAATGAGCATGCTGAGAGCAAAAGAGGCACATCACAATTGAAATAAGGCAGAGAGGGGGACTAATGACAGAAATCTCTCTTCAGTCATATATGTCAGTAGTAAAATGGAATGTTAGCAATATATCAAATGTTTAGAATGATGGAAATGGGATGTTTATATGCCTGTTTAATTATTATTGTTATTTTTACCTTTTTCTCTCTTTTTTCATTTTATCCCACAACTATAACATGTTTACTAAAGAATCTAGTGATTAAAGTTACATAAAGCACTATATAGGACTGTTCTTTTCTAGTTTGTAATTAATGTGTCATCTTTAAATCATCTATCTTTCTGTTTATTGCCTGAGAACTTTATTATTAACATGTTTTTTATATTGTTGTTGTTTTTATTTTTATTATTACTACTATATTATTATCTATTGTCATTGCTACCATAATAATTACTATTATTGTTACTTTTATTCATTATTTATTTTCTTCTTGTTTTTTGCATATAAGGGAATAGAAGGCAAAAGGTCATATATTGAATATTTTTCTGTTCACCTTTTTAAATAATTTGTCATCCCACTAATTCTAGTCATTTACATCATATGATCAGGATTTGATTATTTGATTACTAGTCGTCTTATATTTATTTTTATGTGTGTAATCATGGTTTTAAAACGAACATTCAAATACCTGTCCAAACATTGCAGTATGTTGTATTTATTATATCTAGTGTAATATTTTTCTGTAAATTTTACAATTTATTTAGCAAGCCTCCTGTTTTTATAATTCTATTTTCTGTGTATGGTATATTATGTAATACAGTGTTTTTCTCATTACTTTTTTGGGTTTATAAATTGCACCATCCACCTTATGTAAAGAGTAACTACCATTAAAATGTAACATATATATGCTAATGTCTCCATATATTTTCAGATCATGCAGGCTGTAACAGATGGTAATGGAAACAATGCTGCCTTCACTATGGCTTCAGAAGTCCGCTTTCTGCCACCGTGGGCAAAGTCACAGTTAGCGCACATCTTGGAAGTCACTCATGGCTGGCGGGAGATCATGGGTCGAGTGCCAAGTATGCCATGGGTGCCTGGAGAACCAATCCCAGAGGGTCTTCATTATCCTAGAAAATACACATCTGATGATATTCAGTAAGACTTTGTACCTTGGTCTTGTAATATAATGGATTAATTTTGCTTTATTAAGTATTTGATTGAAGTAGTGTCTTTTTTATGATAAGTTTCAATCAGGCTTCAGGTCTAGTTACTCAGGTATTTAAGTACATAGATCTCAGCTGACTATACACTTCTTTTACTAACATATAAATATGTTGATGCCTTTCTGATAAGGCAATTTCCATGAAGCTAGTCAAGATTCTAGACAAATAATTCTGTTAATGTATGGTCATTTTAACATTTACTTCCATTTTCTCTATGTGTGAGTTCTTATGTTTCATATATGTTTAGTACCTTAATGCAGTGTCTGCCACTAATATGAAAATCTGTTATACTGTTATGTCATTATATCTCAGAGATAGATCATACCTTCTACTACATTTTAGGACTGTCTATATACTCTCATTTTTATGAGGTATTTTGAATGGCAGTGGTTGTCTATATGTGAGCACCCAAACAGTGTTTAGAGATAGTTACTGCTTCCAACTTCAGTTTTAACTTGTCATTGAAATTTATCAAGATGCATTTACTTTTATAAGCATTCAAGTTGGTTGGGTTTACTAGGAATAATAATGCCACAGTGCAGAAGGCTACTTAATCTATGGTAAATGAATAAGGTCTGATGTTTCAGAGTTTGAAAAGGATTGATACTTAAGTAAAGTAAATGTAAATGCAGAAATTGAGGCATATGAGATATGTATTCTCAGTATATGGACAGTATTATTATTTTGTAATTTAAAGTAAGTGATGATACATCTGTTACACATTTTTTGTGAAAAACAGCAGTTCTGCAATTGAAGCACTTTGTCCAGTTTAAGAAATGCCTAAATGTTCTTCCACAGATATGGATGTAATATTAAACCACTAGTGATAAGGCCTTTAACTTTTTAACTTGTCACCTCCATTATTTTTTCTCCTCTTTTTGCGAATTATCTGTTTATGAATTAGTGGAAATGCCATCTCATGTTATGTTAATCAAGATATTTTCTGAAAAGACTAGTAGCTGAAGAATGTGCCCGAGATCGCCGTGAAGGTTTTGAGGTCCTTCTTGAAGAATGGGGAACAAGTGGAAGGAAGAGACCTACGTTGCAAGACCTTGTAAATCTTCTGGAACAGGCAAAACTGTACAGAGCTGTTGATTACTTAACTGTCAAGGTTTTAAATGGTAAGGAGTTACTTTGTTGTTTTAACTAACATTTGTTTGTTTTCTTCTCTATTTCATGCAGATGATGACCCCAGTTGTCTTGCAAGTAGGTCATACATAAAAAGTTAGGCTGTAAGAGATTAAAACATGTACTATCCACTGTTGTTCTTACTGAATTTTGCTGCACAGATGCCTTTTCAAGGATTCAGTTACTGAGTGGTCAATTAGTAGATGCTAGTGACCACCAGATTTCTCCTTTTCCCTAATTTGTAGGAAATTTTGTTTTTCTCTGCTGCTATTATTTTTTATCTGTTATTAATTTTTATTAATATTATTGTGATGTTATTGAAATACTAATAACAATAAAAGACAAAGAGAATCTTTCCAAATATTAGGCAAAAGGATGAACAGGTGAGAAAGGTATGACTGGTGATTGACTCCCTTGATTAATTACTTGTAGAGCTATCAATGTGTAAATGCAATTAATATTCTAAAATACAGTTCTAGAAATATGGGTAATGATAGATTTTAGTTGTTCCAAAGGAATTAGTGAGATCTGCCTTATTGTTAATAGTTATCCATGAATGTATTAATGACCTTAGCTTCTTTTATATGTGAATTTGGTGAAATAACAAAGAGTATACACCATTCTGAAATACATAACTAAAAAAGAATGTTATTCATTAAAGAGAAATCTCATGATTGTTTACAACTATCCCAGTTTAAGTATGTARGTTTATCATTGCCTATATTTTCAGGGGAGCCCCAGAGCAGGGACCAAAGTGAAGGGGAGCTGTTTGATGAGCTAGAGAGAGCAATACAGAATGACCAAAGAGTCCACCAAGACATTGTTCATGGGACCTTCTCTGTTGGGGTGGTCAAAGATACCCCAGACTCACTCTTGACAGACAGGCTTAGTGAGAGATCTGCAGACGATTCTCGAATGCAAGTGAATGAGGAGCCAACCAGAGTGATGGATCTTTCCAGGAACGTTCTAGCTGCGTCACCAGGAAGAGAAGAAATCCAGCAACCGCGATTCAATGCAGAAGTCTTAGAGGGTTTAGATAGTTCAGGCGTTCCACATTTCAGATACTCCATGCTGAAGCAGATCACCCAGAACTTCTCCGACTTACCTTTAGATTATGGTGGAAATAAATTAGGAGAAGGAGCATTTGGAGTGGTCTACTTAGCAAAAATGTATGTTGGTGGGAAAGAGAAAAAGGTCGCAGTCAAGAAGCTGAATTCAGGAGAAGCCAGAGTTGAGCAACAGTTCAAAACAGAGATTGAGATTTTATCAAGGCAAGTAACACATGTTGCATAGTAGAGAAAACATACAGGGTAATTTTATCAAAGCAGAAACACATTGGAGATGGAATAGAGATTTACACTTGATAAACAAAAAAAATATTGGTATCCCTATCCTGGCAGATAACACATAGTAGATAGATGGGAGAAGATACACATCTGACAAATTTAAAATTAATCTGTATCATTGCTTAATATTTTTATAAAAGCAGATCTTTGTTCTTCATTTTTTATTCTTTTTTCTAATGATGTTCTTCTTATTATTCTTTTCTGATCTTATTCTTCTTTTACATTGTTTAGGCCTTTTTATGATGGGCTATTTCTAATGCTTTAATTCTCTCAACAGATGTATACATGAGAACTTATTGCCTTTAGAGGGTTACTCATGTGATGGCCCAGACTGGTGTCTTGTGTACACTTACATGTCTAATGGAAGTCTTCAAGATAGACTTGCTTGCTTAGTAAGTTCAACCAACAAGATTTAAATTTTTCTTCTTTATTATTGTTACACAATATGATAAGCAGAAAGAAATATTTATATATTTAGAAGTTATCTTCATTTTGAAATTGTCAGTTTACATTTTTTTTTATATCAAAGAAGGTACTTATAGAGTATTGATGTTCTAAGATCTAGAATATAGATAAGTATATTTTCTTTTAAGTTTTTTGATAATTTAGGTAGCCAGAGCATTATGTGAGATTAGATTTAAAAGAGGAGTACAGTGTGGTTGGTTCTAGTAGAGGTTATGGAAGTTTATTTAGATAGAACAGGCTGAAAYGGAACTTCTTTCAGAACATTCCAAAATTATCTCTTACTTTTCACTTCCAGAGTGGGACTGAACCTTTAGACTGGACCATGAGAACCCGTATTGGAGAAGGAGCGGCTCGTGGCATAGTGCATCTCCATACATTCCAGGAGCGACCACTGGTTCACCGGGACATTAAAAGTGCAAATATTCTTCTTGATGACAAACTTGTACCTAAGGTTAGCAGAAGAGGGTATTTCTTAATTAGTTACCTTGCATTTTCCTCTTTTTAATGGGGTATTATATTATATTTCTCTTGGTATTGATAAGTCAGAATCTTTATAGTGTACTTTGAAATTGTTCATTTGCAGGTTGGTGACTTTGGTTTAGTGCGTCTAGGAGGCAGCGGCACTCACACTCGCACACTCATCAAAACCACTACAGTCTTTGGCACCTCTGCTTACATGGCGCCAGAAGCTTTTAGAGGAGACATCTCAGTGAAAATGGACACCTTCAGCTTTGGAATTGTATGATTTTATTATTTTTATGTAATAGTTATGTAGAGAGGCAGAAACTGTCCTCATAAGTGAATGAGATGTATATGATATTTTTTCCTCTTTACACTATGGCAAGAATCATAACCACAAAAGTACATGTCAGGTTTTCCAATCCAAATGTATGTATCTTTTAATATTCTTAACTTTTCAGGTTATATTAGAACTCCTCACTGGACTACCATCTTATGATGAGGAACGTGAAGGCTGCGACTTGGTATGTATAATCCCTTTCTTTGGTCAGGAGCATAGCTAGACATTTGTGGGTCAGGTGGATAAGGTCCTTTGAGAGGGTCTATTAATTTGGGGGTTTCATGACCTAAATATGTATGAAAAGATGAAAGTATTTATGTACCCATTTGAACTTAAACATATTTCTGTTCACTTGTGCAAAAAGCATTTGATATCAAAGAAAATTAACCAAATATAAGGTGCACTTCTTGTTGATTAATGTTATTGTAGTTTGCATTGATAGTGGCATTGCCTGCCATCATATTTTGCACTTGGTTATCTCATTATTTTTTTTTACATCTTTGGTTCTCTTTAATATTGATGCTGTTAGAATAGCAAAATATTCTTAAATACCTTGATTTTTTCTTGCATCACAAGCTTAATTTTCCAAAAAGAAAAGATGGAAAAACTTGTATAGGAAATAAACTATAATGAAGTTTAAAAAGAACATGGGTAATGAAATAGGATTTGATAATAAAAAGAAGGAAGACTATCAGGGATGTGTGCTCGGCTCTCCATTTGTATCTTTCTTTTGATTTGAGTAAATTAAATATTTTCTTACACATGTTTATATTTGTTTTTCTCTAATCTTTGTTCTCTTCATTTCCTTTCCACATATTCCTCTTGTTTAAATTTGTAAATATCCTTTTCACAGCTGTCTCATGTCCTGGAAAGTGAAGGGGAAAAGAGTGAATTGCTAGATGTTCGTGCAGGCAGCTGGGACCCAGACATAGCATCTCAGCTCTTTGATTTGGCTGAGCTCTGTACTGATGACAAGAGGAGGCGGCCTACAATGGTTCAGGTGCTCGAAAATTATTCTTCTATTGTACACAGTCAGTAAAATTTAGATGCACT
