# Supplementary material for: The Shrimp NF-κB Pathway Is Activated by White Spot Syndrome Virus (WSSV) 449 to Facilitate the Expression of WSSV069 (ie1), WSSV303 and WSSV371
Source: PLoS One. 2011 Sep 12;6(9):e24773. doi: 10.1371/journal.pone.0024773 (PMC3171479; doi:10.1371/journal.pone.0024773)
Supplement: Figure S2 — A cartoon diagram for vector constructions in cellular localization. (DOC) [file pone.0024773.s002.doc]

**Table S2. PCR primers used in the constructions of luciferase reporter vectors of the 40 WSSV genes that have putative NF-κB binding sites in their promoters**

| **Primers** | **Primer sequences (5’-3’)** | |
| --- | --- | --- |
| **Name** | **Forward** | **Reverse** |
| WSSV052 | GGGGTACCCAATGTGGTAAAAGTGTTTGTTGAT | CCGCTCGAGTTCTTTTGCTTGCTGAGTGTACATA |
| WSSV055 | GGGGTACCTTAGGGGATGGACCGCTG | CCGCTCGAGTATATCAAAAGGGATGGAAACAACT |
| WSSV068 | GGGGTACCTCTTCGAGCCAGAAATACGACATAG | CCGCTCGAGCTGGAACGCTAATGGGTCGC |
| WSSV069 | GGGGTACCAGGGAGATCCTAGAAAGAGGAGTG | CCGCTCGAGCTTGAGTGGAGAGAGAGAGCTAGTTAT |
| WSSV070 | GGGGTACCGAACGCTAATGGGTCGCG | CCGCTCGAGTATCTTCGAGCCAGAAATACGACAT |
| WSSV082 | GGGGTACCTCCTGAGGAAGTAGAGGGCTG | CCGCTCGAGTCTGTATGGGTGATTACGATTCG |
| WSSV084 | GGGGTACCCCTTCATATTCATAAGGGGGG | CCGCTCGAGTATCTGGCCAACCTAAAATGATT |
| WSSV103 | GGTACCCGTGTACAGCCAGATATCTGGAGAG | CTCGAGTGATGTGGCTTTATTCGCCTTC |
| WSSV106 | GGGGTACCCCAATACAACAGAGGCATCTTTACA | GGAAGATCTCTGGGGAATTTCCTCTCCAGA |
| WSV107 | GGGGTACCGTAATATGGATGTATGTACTGCCATGT | CCGCTCGAGGGTGATTATATTTATTAAATATAAACATGAAAG |
| WSSV109 | GGGGTACCATGGACTCTTCTGACTCATCCGAT | CCGCTCGAGTGGGTGTAGAAGACATCCTCGTT |
| WSSV113 | GGGGTACCTACTTAGCCCTGCGTCAATTTC | CCGCTCGAGCCAAGAAGAATGCAAGTGCC |
| WSSV120 | GGGGTACCTGCAACATTTCGAACTCTAGCT | CCGCTCGAGTAAATGGTGATGGAGAAGATTCAGA |
| WSSV205 | GGGGTACCGAAATAGCAGCAGCAGGACTG | CCGCTCGAGTTCTCCATCTCAAAAAATTTTTAAA |
| WSSV212 | GGGGTACCATAGCTCTAATGTCTCCCTTTGTTAC | CCGCTCGAGTCCTTCCTTAAAGATTCTGACAAGG |
| WSSV280 | GGGGTACCTTTGGCGTTCAGTTTAAAAAGAG | CCGCTCGAGTCGAATTGGAACACTCCAGTC |
| WSSV300 | GGGGTACCGAAAACCACTACAAAAGTAAGATGG | GGAAGATCTTAGGTTGGACCAGAAAATGTTACC |
| WSSV303 | GGGGTACCGTTCAGAGATGTCCAGGTCCTTATC | GGAAGATCTCTCGACGTCTGGTCCACTACTG |
| WSSV356 | GGGGTACCGGGAGGGATATTGTTGTTGCTG | CCGCTCGAGCTTCTCATCCAGAAGAGTACTATCAGC |
| WSSV357 | GGGGTACCTCGTCACTCTCTTCATCACTTTCTTC | CCGCTCGAGCCTTCAAATTGTTGTGAGATATGAGAAC |
| WSSV361 | GGGGTACCCATTTCATATCCTCCTCCTGACCT | CCGCTCGAGGCTTTTCGCGCAGGCATTT |
| WSSV370 | GGGGTACCTGTCGTTCATGGCATATTTTCC | CCGCTCGAGGTAATTTGTACGGAGGTATTGATACCG |
| WSSV371 | GGGGTACCTTGAGAATAGACTCGCCGAACAC | CCGCTCGAGGTGCTTGGTGGTAACAGAAAGTACG |
| WSSV377 | GGGGTACCGTGGCCGTCCATCACTCTCTTAT | CCGCTCGAGTCTTCCCAAGAATTTCTCAAATCGT |
| WSSV379 | GGGGTACCAGGTTCCATGTCGGATGAGAG | CCGCTCGAGTGAAAGCAGACGCTCCCTTAC |
| WSSV380 | GGGGTACCGTTGAAGCTGAACAATGTTGTTCC | CCGCTCGAGCAACACTGCTTCAATGGCGAAC |
| WSSV382 | GGGGTACCCATAATTCTTCCCAAATTGTTGTTAT | CCGCTCGAGCGAGATTATTGCTGCCGTCTC |
| WSSV385 | GGGGTACCAATACGCCTGTGAATCTTCCC | CCGCTCGAGGTTCTGGTAGTGTTTTCCCGTTG |
| WSSV418 | GGGGTACCGTGAAAGAAAGATCCATGACGAGT | CCGCTCGAGCTCTTCCACGATGTGGAGTTACAC |
| WSSV421 | GGGGTACCATCAACGACAACAACATAAACTCTTC | CCGCTCGAGGACGAGTTTTTTTCTTTATCGAACG |
| WSSV423 | GGGGTACCTTTCACCCCACATCCACCC | CCGCTCGAGCGACGTTTTTTATACTCTGCTCG |
| WSSV443 | GGGGTACCGTATTGGTTGGTAATATTTTTTCCAAT | CCGCTCGAGTACTGAAAATAATAGAGAGGTGTCTTTTAC |
| WSSV446 | GGGGTACCTGTCTAACGTGGCTACACTCTTTACT | CCGCTCGAGTTACCTAATTTTCTATTATGATAATTATCCTC |
| WSSV464 | GGGGTACCCAGATAAAGACAATGAAATTGCTGTT | CCGCTCGAGTTATTATTTTTAATGTGTACAATAAAAACAATT |
| WSSV466 | GGGGTACCTATTGAACAAAAAATCGAGCCGT | CCGCTCGAGTTCTCTCCAGGAATACTGTTCATGG |
| WSSV467 | GGGGTACCGTTAATGCCGATTTCGGATCAG | CCGCTCGAGCAGGACATCTGGTGATGTGATGTATG |
| WSSV484 | GGGGTACCCTTTTACCCTTTCCAAATCACCAC | CCGCTCGAGTTGGATGAATGTGAAAACTTGCAT |
| WSSV505 | GGGGTACCAGCAGCCGCATTCTGTCTG | CCGCTCGAGGCTCAATCTAAAGGGAAGATGTTACG |
| WSSV507 | GGGGTACCGATTCGGAAGAGTATGATGATGGT | CCGCTCGAGTCTCCATTACATAATAATTTTTAACTCCTTC |
