# Supplementary material for: The Shrimp NF-κB Pathway Is Activated by White Spot Syndrome Virus (WSSV) 449 to Facilitate the Expression of WSSV069 (ie1), WSSV303 and WSSV371
Source: PLoS One. 2011 Sep 12;6(9):e24773. doi: 10.1371/journal.pone.0024773 (PMC3171479; doi:10.1371/journal.pone.0024773)
Supplement: Table S1 — PCR primers used in cellular localization. (DOC) [file pone.0024773.s003.doc]

**Table S1. PCR primers used in cellular localization.**

| **Primers** | **Primer sequences (5’-3’)** |
| --- | --- |
| **Name** |  |
| LvP1(1-129)-Forward | CGGGGTACCCGCCACCATGGAGGCTGTAACAGATGGTAAT |
| LvP1(1-129)-Reverse | CTAGTCTAGAAACCTTGACAGTTAAGTAATCAACAGC |
| LvP2(1-262)-Reverse | CTAGTCTAGAACCATAATCTAAAGGTAAGTCGGAG |
| LvP3(1-536)-Reverse | CTAGTCTAGACTGACTGTGTACAATAGAAGAATAATTTTC |
| LvP4(129-536)-Forward | CGGGGTACCCGCCACCATGGTTAAATGGGGAGCCCCAGA |
| LvP4(129-536)-Reverse | CTAGTCTAGACTGACTGTGTACAATAGAAGAATAATTTTC |
| LvDoral(1-400)-Forward | CGGGGTACCCGCCACCATGGTTGTTGCCCAGCGTACTTCC |
| LvDoral(1-400)-Reverse | AAGGAAAAAAGCGGCCGCCACATATCAGAAAATATCCAAAACTTACC |
